# Combinatorial analyses reveal cellular composition changes have different impacts on transcriptomic changes of cell type specific genes in Alzheimer’s Disease

**DOI:** 10.1038/s41598-020-79740-x

**Published:** 2021-01-11

**Authors:** Travis S. Johnson, Shunian Xiang, Tianhan Dong, Zhi Huang, Michael Cheng, Tianfu Wang, Kai Yang, Dong Ni, Kun Huang, Jie Zhang

**Affiliations:** 1 Department of Biostatistics, Indiana University, School of Medicine, Indianapolis, IN 46202 USA; 2 Department of Medical and Molecular Genetics, Indiana University, School of Medicine, Indianapolis, IN 46202 USA; 3 Guangdong Key Laboratory for Biomedical Measurements and Ultrasound Imaging, School of Biomedical Engineering, Shenzhen University, Shenzhen, 518060 China; 4 Department of Pharmacology, Indiana University, School of Medicine, Indianapolis, IN 46202 USA; 5 Department of Electrical and Computer Engineering, Purdue University, West Lafayette, IN 47907 USA; 6 Department of Pediatrics, Indiana University, School of Medicine, Indianapolis, IN 46202 USA; 7 Department of Medicine, Indiana University, School of Medicine, Indianapolis, IN 46202 USA

**Keywords:** Neurological disorders, Computational biology and bioinformatics, Data mining, Functional clustering, Molecular neuroscience

## Abstract

Alzheimer’s disease (AD) brains are characterized by progressive neuron loss and gliosis. Previous studies of gene expression using bulk tissue samples often fail to consider changes in cell-type composition when comparing AD versus control, which can lead to differences in expression levels that are not due to transcriptional regulation. We mined five large transcriptomic AD datasets for conserved gene co-expression module, then analyzed differential expression and differential co-expression within the modules between AD samples and controls. We performed cell-type deconvolution analysis to determine whether the observed differential expression was due to changes in cell-type proportions in the samples or to transcriptional regulation. Our findings were validated using four additional datasets. We discovered that the increased expression of microglia modules in the AD samples can be explained by increased microglia proportions in the AD samples. In contrast, decreased expression and perturbed co-expression within neuron modules in the AD samples was likely due in part to altered regulation of neuronal pathways. Several transcription factors that are differentially expressed in AD might account for such altered gene regulation. Similarly, changes in gene expression and co-expression within astrocyte modules could be attributed to combined effects of astrogliosis and astrocyte gene activation. Gene expression in the astrocyte modules was also strongly correlated with clinicopathological biomarkers. Through this work, we demonstrated that combinatorial analysis can delineate the origins of transcriptomic changes in bulk tissue data and shed light on key genes and pathways involved in AD.

## Introduction

Alzheimer’s disease (AD) is the most prevalent form of dementia, affecting 40 million people worldwide^1^. Despite decades of research, the etiology of AD remains unclear^2^. The pathological hallmarks of AD include extracellular β-amyloid deposition, tau protein-mediated intracellular neurofibrillary tangles, dystrophic neurites and neurons, synapse death, and proliferation of microglia and astrocytes^2–4^. Microglia, the main immune cells in the central nervous system, typically conjugate and form clusters around amyloid plaques^5,6^. Microglia-mediated neuroinflammation and its potential link to AD etiology are currently a focus in AD research^7–10^. Previous studies of gene expression in bulk brain tissues identified microglia-associated genes and gene networks that were differentially expressed between AD and healthy control tissues^11–15^. Those studies did not account for changes in cell-type composition between AD samples and healthy brain tissues, however. Therefore, it is not clear whether observed changes in gene expression between AD samples and controls are due to transcriptional regulation or to changes in the relative proportions of different cell types in the samples^16^.

It has been proved that changes in overall mRNA levels reflect changes in the cell-type composition of bulk brain tissues affected by AD^16^; however, it is not known how changes in cell-type composition impact the expression of marker genes for specific cell types. Furthermore, it is not clear whether *bona fide* transcriptional regulation can be revealed on top of changes in cell-type composition in bulk tissue samples. To answer those questions, we re-examined differences in gene expression between bulk brain tissues affected by AD and control samples in five large transcriptomic datasets (Table 1), while accounting for changes in the cell-type composition of the samples. We aimed to separate the transcriptomic changes due to transcriptional regulation from that due to changes in cell-type composition. To accomplish that, we performed differential expression (DE) analysis and differential co-expression (DC) analysis on gene co-expression network modules and combined the results with estimates of the relative proportions of brain cell types in the samples.

**Table 1:** Summary of AD and non-dementia control brain transcriptomic datasets used in this study.

	Platform	AD subject	Non-dementia control subject	Brain regions
**Dataset**				
GSE5281 (Liang et al., 2007)	Microarray, Affymetrix HU133	87	74	Middle Temporal Gyrus (MTG), Posterior Cingulate Cortex (PCC), Entorhinal Cortex (EC), Hippocampus (HC), Superior Frontal Cortex (SFG), Primary Visual Cortex (VCX)
GSE48350 (Berchtold et al., 2008)	Microarray, Affymetrix HU133	80	173	EC, Post-central Gyrus (PCG2), SFG, HC
ROSMAP (De Jager et al., 2018)	RNA-seq	224	201	Dorsolateral prefrontal cortex (DLPFC)
MSBB (Wang et al., 2018)	RNA-seq	409	273	Frontal pole (BM10), Superior temporal gyrus (BM22), Parahippocampal gyrus (BM36), Inferior frontal gyrus (BM44)
Mayo^53^	RNA-seq	82	78	Temporal cortex (TCX)
**Validation datasets**				
GSE33000 (Narayanan et al., 2014)	Rosetta/Merck Human 44 k 1.1 microarray	310	157	DLPFC
GSE15222 (Webster et al., 2009)	Sentrix HumanRef-8 Expression BeadChip	176	187	Whole brain with majority of cortex
GSE84422 (Wang et al., 2016)	Microarray, Affymetrix HU133 & HU133_Plus_2	60	65	19 regions
Allen Brain Dementia data (Allen Brain Atlas)	RNA-seq	43	60	HC, temporal cortex (TCX), parietal cortex, forebrain, white matter

Gene coexpresion analysis has been applied in AD research to identify clusters of genes (modules) with specific functions that are dysregulated in AD^15,17,18^. Co-expressed gene modules are often specific to certain cell types^19^. If a co-expressed gene network module specific to certain cell types displays high DE between AD and control samples with little or no DC (change in the correlations between the expression levels of genes with in the module), then the DE is probably due to changes in cell-type composition rather than to transcriptional regulation. By contrast, if the DE is accompanied by DC, then the DE is probably due to transcriptional regulation.

We first performed frequent gene co-expression network (FGCN) mining on five large human AD transcriptomic datasets to identify highly conserved GCN modules across multiple AD cohorts, avoiding potential technical platform or systematic bias in any single study. Then, we computed DE scores and DC scores for each identified gene module and partitioned the modules according to the scores. To adjust for the changes in cell-type composition, we estimated the abundances of neurons, microglia, astrocytes, oligodendrocytes, and endothelial cells in every sample. Our results suggest that DE observed in microglia modules was largely due to increased proportions of microglia in the AD samples rather than *bona fide* upregulation of gene expression within microglia cells. By contrast, DC and DE in neuron modules and astrocyte modules was likely due to the combined effects of altered cell-type proportions and dysregulation of transcription in AD. Furthermore, DE in the astrocyte pathways was highly correlated with clinicopathological biomarkers of AD. We identified six transcription factors that were frequently differentially expressed among nine AD datasets and are predicted to target multiple genes in the neuron modules.

## Results

### Gene co-expression modules were largely specific to either AD or control samples

**Figure 1:**
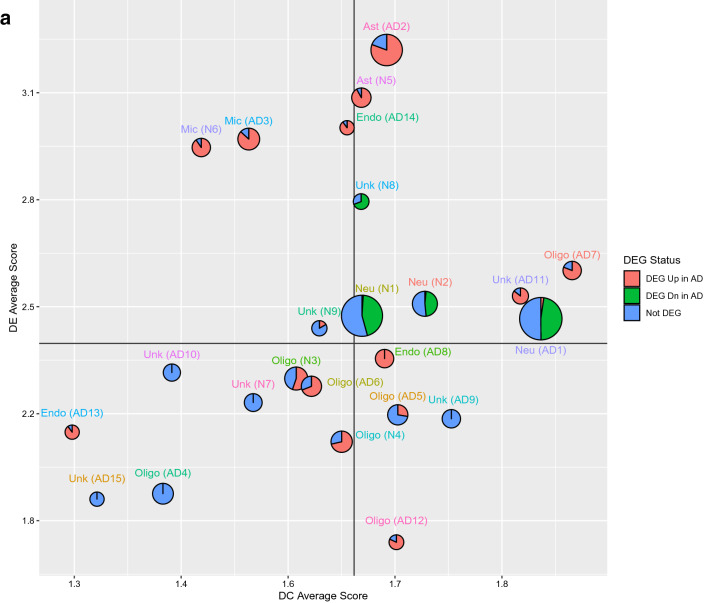

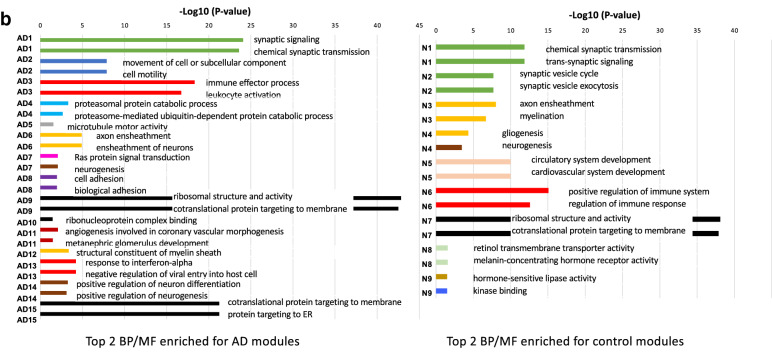

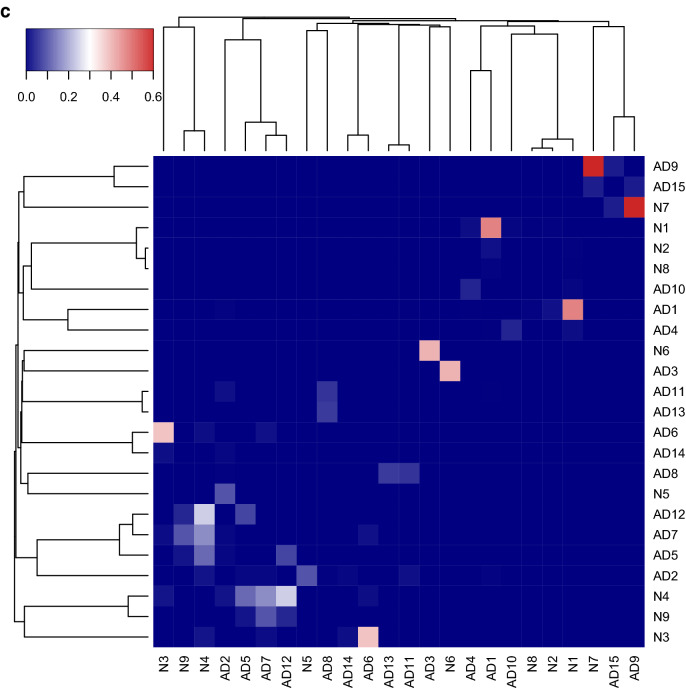

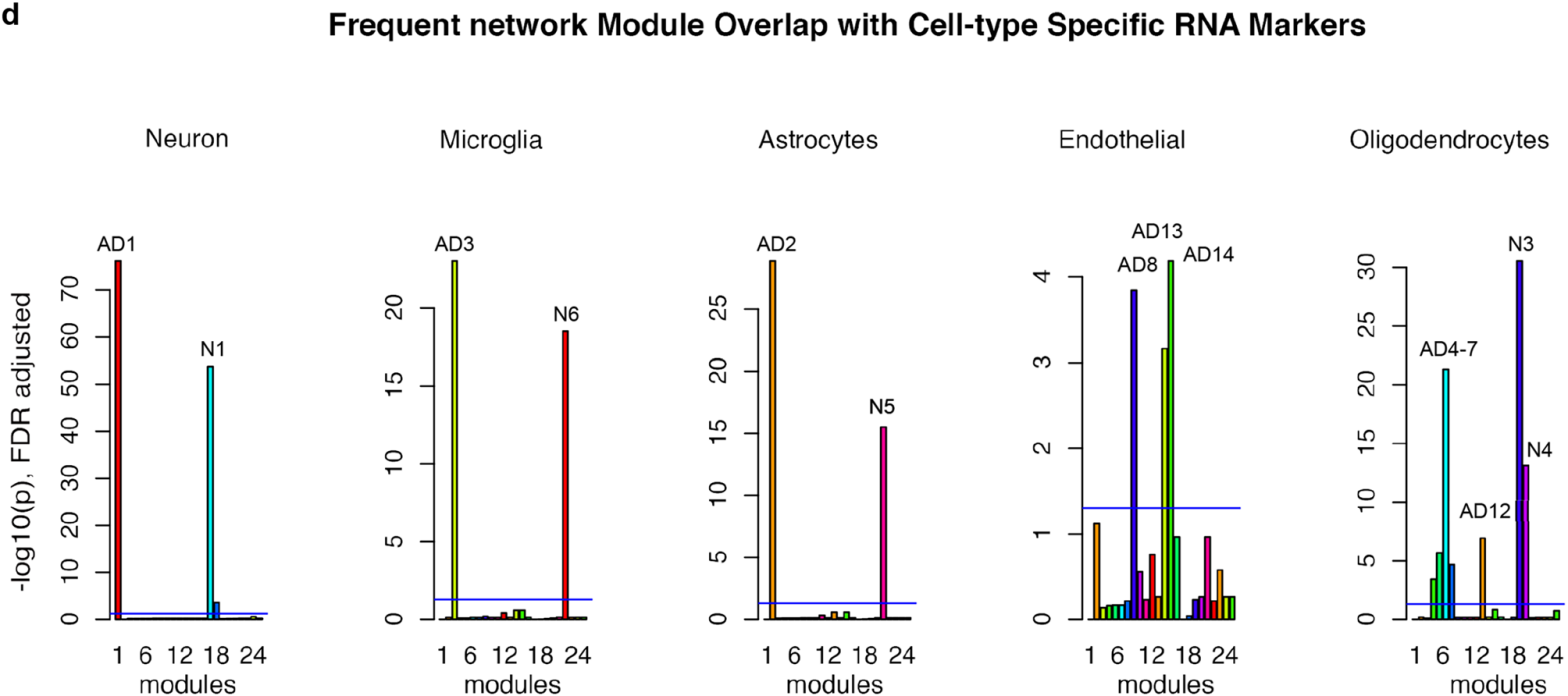
FGCN modules from AD and control samples classified according to differential expression and co-expression levels. (**a**) Differential expression average scores (DE) vs. differential co-expression average scores (DC) of 24 frequent gene co-expression network modules (module names in parenthesis), the majority of which are enriched with specific biological functions as shown in panel b. Most of the modules are also enriched for specific cell-type markers: Neu, neurons; Ast, astrocytes; Mic, microglia; Endo, endothelia; Oligo, oligodendrocytes; Unk, modules not enriched with cell type-specific markers. The pie charts indicate the proportions of differentially expressed genes (up or down in AD vs. control) in the modules. (**b**) The top two enriched biological functions/processes/pathways in each module identified using ToppGene. The colors of the bars are indictive of different biofunctional terms. (**a**). (**c**) A heatmap of the degree of overlap between the genes in each pair of modules mined from the AD and control samples. The modules were rearranged by their similarities and then plotted according to the Jaccard Index values. (**d**) Cell-type enrichment for each module was assessed by cross-referencing the module genes against cell-type signatures of neurons, microglia, astrocytes, oligodendrocytes, and endothelial cells. The x-axis shows the different modules. The 24 FGCN modules are numbered as: 1–15 correspond to modules AD1 to AD15; 16–24 correspond to modules N1 to N9. The significance of the cell-type enrichment in the modules was measured by Fisher’s exact test and corrected for multiple comparisons by the Benjamini and Hochberg procedure. Horizonal blue lines indicate the threshold of false discovery rate 0.05. There is no color match between panels (**a**,**d**).

Gene co-expression network modules are subsets of genes with tightly correlated expression. Genes in a co-expression network are often co-regulated and participate in common biological processes or pathways in homogeneous tissues. Frequent gene co-expression network (FGCN) modules are sets of genes that are frequently co-expressed across multiple independent datasets and therefore unlikely to be artifacts due to systematic biases in cohort composition, experimental design, or technical platforms. We analyzed five large AD transcriptomic datasets and identified 15 FGCN modules in AD samples (Table 1) and 9 FGCN modules in control samples (Supplementary Table 1). We calculated the DE and DC scores between the AD and control samples using a formula modified from Lui et al. 2015 (Supplementary Table 2). We then separated the 24 modules into four categories based on the median DE and DC scores: high DE and high DC (HDC_HDE), high DE and low DC (HDE_LDC), low DE and high DC (LDE_HDC), and low DE and low DC (LDE_LDC; Fig. 1a). The majority of the modules were specific to either the AD or control samples (Fig. 1c; Supplementary Table 2), which is consistent with previous findings that gene co-expression is largely perturbed in AD^15,20^. Only three pairs of modules contained genes that largely overlapped between the AD and control samples (Jaccard index > 0.3; Fig. 1c; Supplementary Table 2).

### The modules were enriched with genes associated with specific cell types and biological functions

We next examined the modules for functional enrichment and cell-type specificity (Supplementary Table 7). We also examined the enrichment of DE genes in each module (pie charts in Fig. 1a). Most of the modules were enriched with genes associated with specific cell types (Fig. 1d) or biological processes (Fig. 1b; Supplementary Table 4). More than half the genes in modules AD1, N1, N2, and N8 were expressed at higher levels in the AD samples than in the control samples, whereas more than 80% of the genes in modules AD5, AD11, and N5 were expressed at lower levels in the AD samples than in the control samples (Supplementary Tables 5 and 6).

The HDE_HDC modules were enriched with neuron (AD1, N1, and N2) and astrocyte (AD2 and N5) markers and functions linked to AD pathology. Modules AD1, N1, N2 were enriched with genes involved in neuronal function^21^. Modules N5 and AD11 were enriched with genes involved in angiogenesis and vasculature development, which is consistent with the finding that changes in vasculature leading to altered blood flow in the brain are a risk factor for AD^22–24^. Module AD2 was enriched with genes linked to gliogenesis and cell motility. Module N8 was enriched with genes involved in gap junction, which supports the hypothesis that disruption of gap junctions associated with the blood–brain barrier in AD allows viruses and bacteria to access the brain^25–28^.

The HDE_LDC modules were enriched with microglia (AD3, N6) and endothelial (AD14, AD8) markers. The majority of genes in those modules were expressed at higher levels in the AD samples than in the control samples and had functions related to immune response (modules AD3 and N6), neurogenesis (module AD14), or cell adhesion (module AD8).

The LDE_LDC and LDE_HDC modules were enriched with oligodendrocyte markers. The LDE_HDC modules were enriched with genes involved in gliogenesis (AD7), ribosomal function (AD9), and myelination (AD12), whereas the LDE_LDC modules mainly contained genes with housekeeping functions such as translation (AD10, AD15, N7), interferon signaling (AD13), protein metabolism (AD4), lipid metabolism (N3), and gliogenesis (N4, AD6). None of the LDE_LDC modules except for AD6, AD12, N3, and N4 contained genes that were differentially expressed between the AD and control samples. Those result suggest that modules with low DE are not strongly associated with AD pathology.

### The proportions of neurons and glia were reduced and increased, respectively, in the AD samples compared with those in the controls

**Figure 2:**
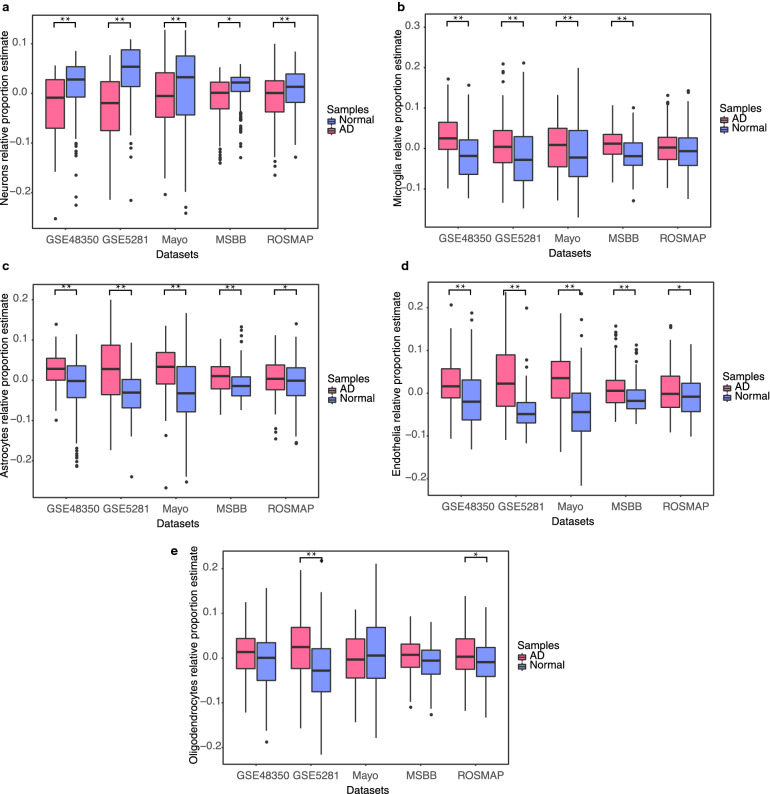
Cell type deconvolution analysis indicated decreased neuron proportions and increased glia proportions in the AD samples. The significance of cell-type proportion changes between AD samples and controls was measured by Wilcoxon rank sum test (*p < 0.05, **p < 0.01). (**a**) The estimated neuron relative proportions in AD samples and controls. (**b**) Estimated microglia relative proportions in AD samples and controls. (**c**) Estimated astrocyte relative proportions in AD samples and controls. (**d**) Estimated endothelial cell relative proportions in AD samples and controls. (**e**) Estimated oligodendrocyte relative proportions in AD samples and controls.

There is mounting evidence of increased gliosis and reduced proportions of neurons in brain tissues affected by AD in comparison with healthy brain tissues^29–31^. We estimated the relative numbers of five major brain cell types in AD and control brain samples using BRETIGEA^32^. The results showed that compared with the control samples, the AD samples across all five datasets had reduced numbers of neurons and increased numbers of microglia, astrocytes, and endothelia (Fig. 2), which is consistent with previous reports^4,30,33^. The relative numbers of oligodendrocytes differed between the AD samples and the controls in only two of the datasets.

### Differential expression in microglia modules was largely due to changes in cell-type composition

Our combined DE-DC analysis revealed that the overall gene expression changes in the HDE_LDC modules were likely the result of changes in cell-type composition between the AD and the control samples. The HDE_LDC modules included N6, AD3, and AD14 (Fig. 1a); however, because of the relatively high DC value of AD14, we focused our further analysis only on AD3 and N6. Seventeen of the 21 genes in module N6 were also in module AD3. The two modules’ summarized expression (eigengene) correlated very highly and consistently in both AD and control cohorts through all five datasets (Supplementary Table 18), so we combined the two modules into one, which we refer to as the microglia module.

**Figure 3:**
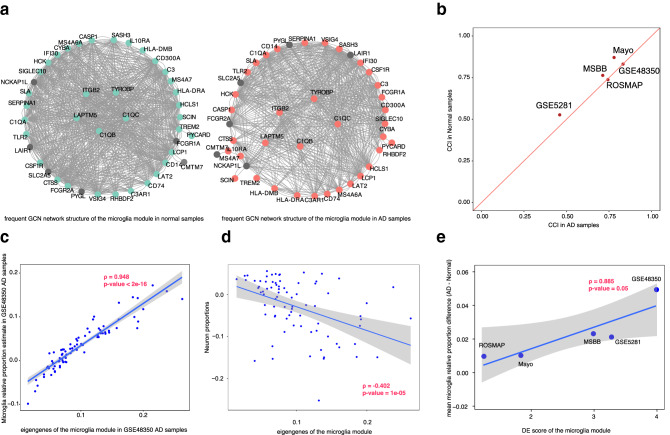
The high differential expression in microglia modules with low differential co-expression was largely due to changes in cell-type proportions. (**a**) Network visualization of the microglia module in the AD samples and controls (for legibility, only gene links that appeared in at least in four of the five datasets are shown). The genes with blue color in control networks and red color in AD networks were had higher expression in the AD samples than in the control samples in at least two of the five datasets. (**b**) Centered concordance index (CCI) values of the microglia module in AD samples vs. controls in each of the five datasets. (**c**) The Pearson correlation between microglia module eigengenes and the estimated microglia relative proportions in the GSE48350 dataset. (**d**) The Pearson correlation between microglia module eigengenes and the estimated neuron relative proportions. This is shown as a poor correlation to compare with panel c. (**e**) The differential expression scores of the microglia module with the mean microglia relative proportion changes between the AD samples and controls across the five datasets. The gray area indicates the region defined by 95% confidence intervals.

The microglia module was enriched with innate immune-system functions and several infectious-disease pathways, but it did not contain cytokine genes (Supplementary Fig. 1, Supplementary Table 2). Nearly 90% of the genes in the microglia module were upregulated in the AD samples in at least two of the five datasets (Fig. 1a). Notably, genes in this microglia module are also among the core microglia genes noted in a previous study^10^, and one third of this module (14 out of 42) are also identified from a microglia subpopulation from a single cell transcriptomic study that is highly correlated to AD pathology^34^. As shown in Fig. 3a, the co-expression network density of the microglia module was high in both the AD samples and the control samples.

To verify the co-expression among genes within the microglia module, we used the centered concordance index (CCI)^35^ to evaluate the gene inter-correlation of the microglia module in each of the five datasets separately. The CCI values indicated that the genes were highly correlated with each other in both the AD samples and the control samples in all five datasets (Fig. 3b). Furthermore, the microglia module expression, as measured by the module’s eigengenes, was strongly correlated with the estimated microglia proportions in the samples [Pearson Correlation Coefficient (PCC) = 0.948; Fig. 3c for GSE48350; Supplementary Fig. 2 for the other four datasets]. In comparison, there was much less correlation between the eigengene values of the microglia module and the neuron proportions in the samples (Fig. 3d for GSE48350; Supplementary Fig. 2 for other four datasets). There was a strong positive correlation between the DE scores and the microglia proportions in the samples (PCC = 0.885, *p* value < 0.05; Fig. 3e). Those results confirmed that the co-expression of the microglia core genes was not perturbed in the AD samples, and that the relatively high expression of the genes in the microglia module in the AD samples was mostly due to the high numbers of microglia cells in those samples.

We also examined the two microglia modules AD3 and N6 separately for their module expression with respect to microglia proportion among AD and control cohorts in all five datasets. The high correlation of module eigengene to microglia proportion (PCC) is very close for the two modules regardless in AD or control cohorts (Supplementary Table 18), which further confirmed that the two microglia modules expressions mostly reflect the corresponding microglia proportion in the same way among AD and control samples.

### Differential expression in neuron modules was likely due to both decreased numbers of neurons and regulatory changes related to neuron hyperactivity

**Figure 4:**
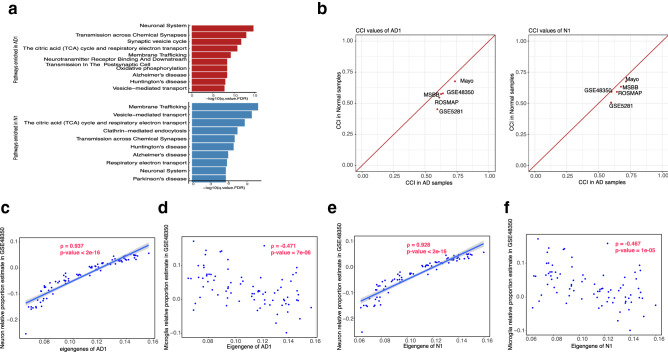
Neuron modules high differential expression and high differential co-expression that may be due to both cell population change and regulatory changes. (**a**) Functional enrichment in the AD1/N1 modules. (**b**) Centered concordance index values of the AD1/N1 modules in AD samples vs. controls in each of the five datasets. (**c**) In the GSE48350 dataset, the Pearson correlation between module AD1 expression (eigengene values) with the estimated relative proportions of neurons. (**d**) The Pearson correlation between AD1 eigengene values and the estimated microglia relative proportions in the GSE48350 dataset. (**e**) The Pearson correlation between N1 eigengene values and the estimated neuron relative proportions in the GSE48350 dataset. (**f**) The Pearson correlation between the N1 eigengene values and the estimated microglia relative proportions in the GSE48350 dataset. This is shown as a poor correlation to compare with panel (**e**).

The HDE_HDC modules had highly altered gene expression and co-expression between the AD and control samples, indicating regulatory changes are involved. We conducted further analysis of AD1 and N1, which were the largest modules in the AD and control samples, respectively, and contained many of the same genes (Fig. 1c; Supplementary Fig. 3). Both modules were highly enriched with markers of neuronal functions, energy metabolism, and mitochondrial functions (Fig. 4a). We categorized the genes in those modules into three groups based on their presence in either or both modules (Supplementary Fig. 3). The results of gene ontology (GO)/pathway enrichment analysis for the three groups of genes are shown in Supplementary Tables 8, 9, and 10. The genes shared by both modules were enriched in pathways related to AD pathology, synapse, the citric acid cycle, and respiratory electron transport. AD1 was more enriched than N1 with genes involved in protein–protein interactions at synapses, glutamate binding, activation of AMPA receptors and synaptic plasticity, and voltage-gated channel activity. Genes involved in ubiquitin-mediated proteolysis were present only in N1. The functions of the genes unique to AD1 are in accordance with the recent discovery of neuron hyperactivity in brains affected by AD^36,37^. The unique presence of genes involved in ubiquitin-mediated proteolysis in N1 suggests that control of the ubiquitin-mediated proteolysis pathway is disrupted in AD. Such disruption has been observed in other neurodegenerative disorders and has been attributed to filamentous protein aggregation in the brain^38^.

**Table 2:** Neuron modules AD1/N1 are predicted to be targeted by differentially expressed TFs.

Module name	Enriched TF	Differential expression in AD versus control	Number of predicted target genes in the module	Fisher’s exact test adjusted *p* value
AD1	BCL6	Up in 7/9 datasets	119/1247	0.0095
	JUND	Up in 4/9 datasets	116/1247	0.0148
	ZBTB16	Up in 5/9 datasets	128/1247	0.0200
	STAT3	Up in 7/9 datasets	204/1247	0.0449
	MYB	Down in 4/9 datasets	135/1247	8.87E-05
N1	ZBTB16	Up in 5/9 datasets	112/1003	0.0058
	BCL6	Up in 7/9 datasets	97/1003	0.022
	LEF1	Up in 6/9 datasets	273/1003	0.0337
	STAT3	Up in 7/9 datasets	160/1003	0.0432
	JUND	Up in 4/9 datasets	94/1003	0.0359
	MYB	Down in 4/9 datasets	113/1003	6.271e-05

Almost half the genes in each neuron module had lower expression in AD samples than in control samples in at least two datasets (Fig. 1a**;** Supplementary Table 1). To investigate how the expression of the neuron modules was affected by changes in the proportion of neurons, we checked the correlation between the AD1/N1 eigengenes and the proportions of neurons across all samples. The expression of AD1 and N1 was highly correlated with the proportions of neurons in all five datasets (median PCC value of 0.937 for AD1 and 0.928 for N1; Fig. 4c,e for the GSE48350 dataset; Supplementary Figs. 4 and 5 for the other four datasets). In comparison, correlations were much weaker between the neuron module expression and the proportions of microglia cells (Fig. 4d,f for GSE48350; Supplementary Figs. 4 and 5 for the other four datasets).

The high DC scores of the neuron modules suggested large changes in gene co-expression in AD. The CCI values were consistently higher in the AD samples in all five datasets (Fig. 4b), indicating that the genes in the neuron modules were more tightly co-expressed in the AD samples than in the control samples. Because three of the five datasets contained multiple brain regions, we first checked if the proportions of neurons across the different brain regions varied more in the AD samples than in the control samples, which could potentially result in high correlations of gene expression profiles among multiple regions. AD is first observed in the hippocampus region and gradually spreads to the entire cortex^39^. The extent of neuron loss might reflect the successive spread of the disease across the different regions, but the difference in neuron loss might not exist in healthy aging brains. We used the Mount Sinai Brain Bank (MSBB) dataset, which contained data from the frontal pole, the superior temporal gyrus, the parahippocampal gyrus, and the inferior frontal gyrus, the inter-correlation of neuron proportions across the four brain regions (CCI = 0.820) was actually lower in the AD samples than in the controls (CCI = 0.944). For comparison, the inter-correlation of microglia relative proportions across the four brain regions was comparable between the AD samples and the controls (CCI = 0.856 for AD, 0.868 for control). We therefore concluded that the variation of neuron relative proportions across brain regions in the AD samples did not contribute significantly to the high DC values in the AD1 and N1 modules.

### Structural changes in the GCN of the neuron modules suggest that differential expression in those modules was due to regulatory changes

We further examined structural changes in the gene co-expression networks by checking for the loss of hub genes (defined as genes in the top fifth percentile in terms of network connections). We obtained 547 hub genes each separately in the AD samples and the controls. Then, we determined which hub genes were lost or gained in the AD samples. We identified 136 genes for each case (Supplementary Fig. 6; Supplementary Table 14). Most of those genes were present in the neuron modules, which suggested extensive changes in the gene co-expression network connections of those modules. The network connectivity among the altered hub genes was negatively correlated with the proportions of neurons in both the AD samples and the control samples (Supplementary Fig. 12; sign test *p* value = 3.24×10^–83^ for AD hub genes, 1.78×10^–27^ for control hub genes). Those results were in agreement with the high CCI values for the neuron modules in the AD samples (despite lower neuron proportion) (Fig. 4b) and indicated that the inverse relationship between network connectivity and the proportion of neurons was probably not a reflection of AD-specific pathological changes, but rather a brain connectome property related to normal neuron depletion due to aging.

In the AD1 module, the gained and lost hub genes included multiple transcription factors. *PEG3*, *ZNF365*, *HERC1*, *FBXW7*, *TERF2IP*, *STAT4*, *NLK*, *RBFOX2*, *CDK5*, *POLR2K*, *PSMD12* were gained in the AD samples, whereas *BZW2*, *RNF6*, *ZBTB11*, *SORBS3*, *TRIM56*, *MORF4L1*, *CNOT7*, *RBCK1*, *SMYD3*, *RAN*, *KLHL12*, *ZC3H15*, and *EID1* were lost in the AD samples. The network structural changes and transcription factor changes not only indicated that the DC was a *bona fide* correlational change among module genes, but also suggested that the DE observed in the two neuron modules reflected regulatory changes.

### Multiple transcription factors were differentially expressed for the neuron module genes

To further confirm the expression regulation changes among the neuron modules, we performed regulatory transcription factor-enrichment analysis to identify regulatory cascades within each module using the “TRANSFAC and JASPAR PWMs” database^40^. Many genes from the neuron modules were targeted by transcription factors that were frequently differentially expressed in the five datasets, as well as in four additional validation datasets (Table 2). Specifically, five transcription factors were predicted to regulate 116 to 204 genes in AD1, and six transcription factors were predicted to regulate 94 to 273 genes in N1 (Supplementary Table 11). To verify that the transcription factors regulate the predicted genes, we acquired the ChIP-seq peak data of the top differentially expressed transcription factors, Bcl6 and Stat3^41,42^. We then crosschecked AD/N1 gene promoter regions for binding peaks for those two transcription factors. We found Bcl6 binding sites in the promoter regions of 18 genes in AD1 and 56 genes in N1. Similarly, we found Stat3 binding sites in the promoter regions of 238 genes in AD1 and 685 genes in N1 (Supplementary Table 12). Bcl6 is a transcription repressor that interacts with the Stat protein to modulate gene expression in B cells^43^. It also plays a role in neurogenesis and neuron differentiation^44^. Evidence from animal models indicated that constitutive expression of *BCL6* in transgenic mice upregulated the expression of *ANXA6, MAPK6, HDAC9, PFKM, GABARAPL1*^45^, all of which were present in the AD1 module. Constitutive *BCL6* expression in a BCL6-activating mouse strain^46^ was also shown to downregulate the expression of *CCND2* and *CDKN1B*, both of which were present in the N1 module. The Stat3 protein upregulates *ATR* expression to control DNA damage and apoptosis. It also binds to the promoter region of *OPA1* and upregulates *OPA1* expression^47^. *OPA1* is essential for mitochondrial fusion, and its mutation has been associated with neurodegenerative diseases^48^. *OPA1* and *ATR* were both present in the AD1 and N1 modules.

The enrichment of differentially expressed transcription factors and their confirmed regulatory roles in the AD1 and N1 genes suggests that DE in those modules was due not only to neuron loss but also to upstream regulatory changes.

### In the astrocyte, oligodendrocyte, and epithelial modules, changes in cell-type proportions contributed partially to the observed differential expression

For other HDE_HDC modules, the two astrocyte modules (AD2 and N5) showed high DC and the highest degree of DE of all the modules (Fig. 1a). There was no correlation between the change in astrocyte proportions in the samples and the DE scores of the astrocyte modules across the five datasets [PCC = -0.06 (AD2), -0.13 (N5)]; Supplementary Figs. 10 and 11). We also found that the astrocyte proportions were highly correlated with the AD2 and N5 eigengenes within each dataset [PCC = 0.846 ± 0.060 (AD2), 0.896 ± 0.052 (N5)]. Therefore, the poor correlation between the DE scores in the astrocyte modules and astrocyte proportions in the samples was likely due to both heterogeneity of the astrocyte proportions across the cohorts and a regulatory effect on the module genes. The CCI plots (Supplementary Fig. 9A and 9D) confirmed that the astrocyte modules exhibited high correlational changes between the AD and control samples in all five datasets, suggesting that regulatory changes were involved.

For the oligodendrocyte module AD7 and the endothelial module AD14, changes in cell-type composition had little effect on expression levels, probably because the changes in the proportions of those cell types were either minimal (for oligodendrocytes) or heterogeneous (for endothelia). The DE scores for those modules were poorly correlated with the cell-type proportions (PCC -0.37 for AD7, 0.74 for AD14; Supplementary Fig. 11). Furthermore, the correlations between the cell-type proportions and the AD7 and AD14 eigengenes were relatively weak [PCC 0.726 ± 0.036 (AD7), 0.418 ± 0.254 (AD14); Supplementary Fig. 10]. The CCI values were different between the AD samples and controls in all five cohorts (Supplementary Fig. 9B and 9C), which implies that module-level correlational changes were more evident in the oligodendrocyte and endothelial modules than in the microglia module.

Taken together, the results indicated that in contrast to the situation in the microglia module, a substantial regulatory effect was responsible for DE and DC in the astrocyte, oligodendrocyte, and epithelial modules.

### The modules with high differential expression were associated with AD neuropathological features and cognitive deterioration

**Figure 5:**
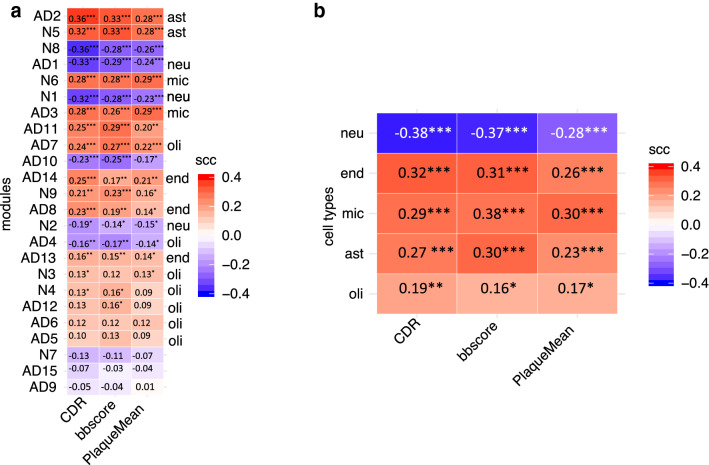
Modules with high differential expression are associated with Alzheimer’s neuropathology and cognitive status. (**a**) Spearman correlation coefficients (SCCs) between module eigengene values and three major clinicopathological attributes: clinical dementia rating (CDR), neurofibrillary tangle burden (Braak & Braak score), and beta-amyloid burden (PlaqueMean). The modules are ranked by the mean SCC values and marked by corresponding cell types: ast, astrocytes; mic, microglia; neu, neuron. (**b**) Correlation between brain cell-type relative abundance and clinicopathological attributes. The brain cell types are ranked by the mean scc value. ast, astrocytes; mic, microglia; neu, neuron; end, endothelia; oli, oligodendrocytes. The asterisks indicate the significance associated with each SCC. *p < 5.7 × 10^–4^ (significance cutoff after Bonferroni correction), **p value between 10^–5^ and 10^–8^, ***p < 10^–8^.

Because the modules with high differential expression were enriched with neuronal and microglia functions, pathways, and genes previously linked to AD pathology, we examined their associations with clinicopathological features. We computed the correlation between the module eigengenes and the Clinical Dementia Rating scale (CDR), the Braak & Braak stage score (BB score; measures neurofibrillary tangle), and the beta-amyloid deposition plaque scale (Plaque_Mean) in the MSBB dataset. We then ranked the modules according to the average strength of correlation with the three attributes (Fig. 5a).

The modules with high DE showed stronger correlations with the clinicopathological markers than the modules with low DE (Fig. 5a). The expression of the astrocyte modules was most strongly correlated with the three AD clinical biomarkers, which is consistent with previous findings that astrocytes are involved in the sustained immune response and mitochondria-related function in brains affected by AD^4,13^. The expression of the neuron modules, AD1 and N1, was negatively correlated with all three clinical attributes, which is consistent with a gradual loss of neurons and neuronal functions in AD progression. The expression of the two microglia modules was positively correlated with all three clinical attributes, which is consistent with the activated immune response observed in brains affected by AD. The expression of the endothelial modules (AD8, AD13, and AD14) was weakly and positively associated with the clinical attributes. The expression of the oligodendrocyte modules (AD4, AD5, AD6, AD7, N3, and N4) was even more weakly associated with the clinical attributes. Of all the modules whose expression was more than moderately correlated (correlation coefficient absolute values > 0.3) with the clinical attributes, only N8 and AD11 were not specific for certain cell types (Fig. 5a; Supplementary Table 15). Module N8 was enriched with gap junction functions and was negatively correlated with all three clinical attributes, which is consistent with the gap junction dysfunction observed in AD^49^. Module AD11 was enriched with angiogenesis functions and was positively correlated with CDR and BB stage, which is consistent with the role of neurovascular dysfunction in AD^22^.

We also analyzed the correlations between the cell-type proportions in the MSBB dataset and the three clinical attributes to determine whether changes in specific cell-type proportions were correlated with disease progression. We found that the proportions of each cell type, except for oligodendrocytes, were highly correlated with all three clinical attributes (*p* < 10^–9^ for each correlation; Fig. 5b; Supplementary Table 15). The proportions of neurons were negatively correlated with the three clinical attributes, which is consistent with gradual neuron loss in AD progression^21^. The proportions of microglia and astrocytes were positively correlated with the clinical attributes, which is consistent with microgliosis and astrogliosis around amyloid plaques^4^. We also observed a positive correlation between the proportion of endothelial cells and AD progression, which is consistent with endothelial activation in AD^23,24,50^.

## Discussion

Next-generation sequencing has generated many large transcriptomic datasets from tissues of mixed cell types for AD research^51–53^. Single-cell sequencing techniques have started to emerge; however, issues such as the technical difficulty of cell dissociation and sample preparation, high dropout rate for signal reading, and scarcity of brain samples make it unlikely that large cohorts of single-cell AD data will be available in the near future^34^. Comparative studies of AD and control samples in bulk tissue transcriptomic datasets have been conducted to take full advantage of those datasets^15,18^. Some studies also applied a gene co-expression network mining approach to single datasets. For example, Zhang et al. applied weighted gene co-expression network analysis (WGCNA) and identified an AD-associated module enriched in microglial function^15^. In another study, Miller et al. adopted WGCNA to uncover disease-relevant expression patterns for major cell types^54^. However, these studies all focused on just a single dataset. In this study, we analyzed FGCNs in five independent transcriptomic datasets encompassing a total of 1,681 tissue samples to avoid potential bias from using a single dataset. We identified co-expressed gene modules that were consistently present in multiple brain regions. The member genes in each module were consistently correlated with each other across all five datasets.

Many transcriptomic analyses have shown significant upregulation of immune response genes and downregulation of neuronal genes in AD^15,18^. It is widely accepted that microglia cluster near amyloid plaques and that microglia-mediated inflammation is activated in AD^5,55,56^. Most previous studies of bulk brain samples containing mixed cell types did not account for differences in the relative abundances of microglia cells in the samples. To fully and accurately infer AD-associated transcriptomic changes, we re-examined five AD transcriptome datasets to determine whether transcriptomic changes observed in those datasets are due to changes in gene regulation, cell composition, or both.

FCGN modules enriched with microglial markers showed high DE and minimal DC between AD samples and controls. The proportion of microglia cells was higher in the AD samples than in the controls. Together, those results indicate that the DE in those modules was largely due to an increase in the proportion of microglia in the AD samples rather than to a change in transcriptional regulation, which is consistent with previous findings^16^. That does not rule out the possibility of a small subpopulation of disease-associated microglia (DAM) in brains affected by AD^34,57,58^. Pro-inflammatory and anti-inflammatory genes and DAM signature genes were previously identified in an *APP* transgenic AD mouse model^58^, which may be more similar in terms of disease mechanism to early-onset AD than to the late-onset AD analyzed in our work. In addition, the previously identified DAM-related genes do not include the mic1 gene cluster identified in a recent single-cell RNA-seq study of late-onset human AD^34^ (Supplementary Table 13). Of the 77 DAM-related mic1 genes identified in the single-cell RNA-seq study, 14 were co-expressed in the core microglia gene module identified in our study (AD3/N6; enrichment *p* = 4.38 × 10^–24^; Supplementary Table 13). The CCI of that subset did not change when we analyzed those 14 genes separately (Supplementary Fig. 7). Moreover, nine ribosomal genes among the 77 DAM-related genes were identified in ribosomal modules (AD9, AD15, and N7), and four more were scattered in modules AD1, N1, and AD13. We did not find the rest of the previously identified DAM-related genes in any FGCN module. This could be due to the following reasons: (1) because all five datasets included multiple brain regions, each gene expression level was averaged across all regions prior to our co-expression analysis. The proportion of DAM cells in brains affected by AD is low (0.6%)^34^, so any elevated expression of DAM-related genes might have been averaged out, or the expression variances might not have been high enough to be detected. (2) It is also possible that because of the cutoff of 10 genes for the minimal size of an FGCN module, DAM-related genes that were not tightly co-expressed with more than nine other genes were not identified.

To further investigate whether the DAM module^34^ could be captured in transcriptomic data from bulk tissue samples, we reconstructed a synthetic module with the mic1 genes (Supplementary Table 13) and analyzed its expression (eigengene) with respect to cell-type proportion changes and DE scores (Supplementary Fig. 13). The low correlation between the synthetic module expression and the change in microglia proportion (PCC = 0.28) suggests that the DAM subtype is not well presented in bulk tissue transcriptomic data (Supplementary Fig. 13A). Consistent with that finding, the DE scores of the synthetic module across the five datasets showed weaker correlation (PCC = 0.7) with the microglia proportions than the DE scores of the AD3/N6 microglia module (PCC = 0.95; Supplementary Fig. 13B).

The microglia core gene module did not include any cytokine genes, which are known to be upregulated in AD brains^59^. We cannot not rule out the possibility that individual microglia genes, such as TREM2 or TYROBP, are upregulated in some AD samples, as observed previously^11,60,61^. However, in the five datasets we analyzed, the DE in the tightly co-expressed core microglia module as a whole was largely due to microglia proliferation in AD.

Although the neuron modules AD1 and N1 shared many common genes, many of the hub genes changed in AD, and the connectivity of the hub genes was negatively correlated with the neuron proportions (Supplementary Fig. 12). Several transcription factors that are known to be differentially expressed in AD might account for such changes (Table 2). The DE of those transcription factors in AD was further confirmed in four additional transcriptomic datasets (Tables 1 and 3). To verify that the transcription factors regulate the genes in the neuron modules, we analyzed Bcl6 and Stat3, the most frequently differentially expressed transcription factors in our AD datasets. We found that Bcl6 and Stat3 bind to a large number of genes in the neuron modules, as indicated by ChIP-seq experiments, which provides strong evidence that they serve as upstream regulators of gene expression in those modules.

In addition to experimental evidence from transgenic mice that Bcl6 and Stat3 regulate M1 genes, we found that 21 of the genes in module AD1 are Stat3 upstream regulators, eight of which were recently shown in transgenic mouse studies to be involved in AD pathology (*MAPK1*^62^, *RHEB*^63^, *SUMO1*^64^, *VPS35*^12^, *GSK3B*^65^, *DYRK1A*^66^, *EPHA4*^67^) or in Parkinson’s disease (*PAK4)*^68^. Recently, Stat3 inhibition was shown to inhibit human astrocyte differentiation and promote neural progenitor-cell differentiation^69^ and also to ameliorate astrogliosis in AD model mice^70^. Combined with the fact that Bcl6 regulates the expression of *STAT3*^71^, those results further support a central role of Stat3 in the regulation of the neuron modules.

Although the overall expression of the neuron modules was lower in the AD samples than in the control samples, the overall co-expression level of the neuron modules was higher in the AD samples than in the controls in all five datasets. The stronger co-expression in the neuron modules might be linked to neuronal hyperactivity in AD, as previously observed in human and mouse models^36^. Evidence from imaging and molecular studies revealed that neuronal hyperactivity, especially glutamate signaling, occurs in the cortex and hippocampus during or even before mild cognitive impairment, which modulates A$\upbeta$ levels and triggers synaptic dysfunction in AD^36,37,72^. Experiments in mouse models of AD showed clustering of hyperactive neurons near amyloid plaques and, furthermore, that reduction of neuronal hyperactivity can prevent build-up of amyloid plaques and synapse loss^73–76^. Because many genes that uniquely presented in the neuron modules in the AD samples are involved in glutamate signaling and synaptic activity (see Supplementary Table 9), we speculate that the strong co-expression among those genes, as well as the upregulated transcription factors, means that some form of neuron hyperactivity still remains in brains affected by AD. Previous studies indicated that such hyperactivity was only present in the asymptomatic phase of AD^77,78^. We hypothesize that such signals might previously have been overlooked because of the overall decrease in the proportion of neurons during late-stage AD. In the future, we plan to further quantify the impact of the neuron population change on expression signals and to mitigate its effect in order to discover true regulatory changes.

Compared with the controls, the AD samples showed low expression for neuron modules and high expression for microglia, astrocyte, and endothelial modules. That is consistent with a recent single-cell transcriptomic study that showed that changes in gene expression in AD are highly cell-type specific, with most altered expression occurring in neurons or a specific glia types^34^. Our results show that changes in cell-type abundances play a major role in determining the DE of microglia and neuron genes between AD samples and healthy brain samples.

Astrocytosis in the AD samples might partially account for the DE in astrocyte modules within each dataset, which is in agreement with the previous findings that astrocytosis is a characteristic of AD^4^. Unlike the microglia modules, the astrocyte modules exhibited DC, which suggests that a regulatory mechanism underlies DE in astrocytes during AD. It is worth noting that the astrocyte modules exhibited the strongest correlation with clinical disease attributes (Fig. 5a).

Oligodendrocyte proportions were not ubiquitously changed between the AD samples and controls across the five datasets (Fig. 2). Their proportions also had the weakest correlation with DE scores, module expression, and clinicopathological markers. In contrast, the proportions of endothelia differed between the AD samples and controls, but, like the oligodendrocyte proportions, they did not correlate with DE in the modules, which suggests either that the proportional changes were inconsistent among AD samples or that transcriptional regulation contributed more than the cell-type proportions to DE in those modules (Supplementary Figs. 9, 10, and 11).

Our study had certain limitations. First, the transcriptomic data we analyzed did not contain microRNA information. Therefore, the FGCN module mining and DE/DC analysis did not include those types of molecules and their potential regulatory relationships. Second, the transcriptomic datasets were generated from tissue mixtures from multiple brain regions, and the expression levels of each gene were averaged across the tissues from all regions. That might be the reason that the previously identified DAM species were not captures in our analyses. In addition, the minimum module size for an FGCN was 10, which might have caused some core microglia genes and DAM genes to be left out of the FGCN modules. Third, it is possible that the total amount of RNA per cell changes when the cells undergo a phenotype change. However, based on the available transcriptomic data, and the fact that no universal nucleic RNA quantification method is currently available (some studies may be conducted for neurons, but we are not aware of such research for other cell type and subtypes), we could take into account changes in the amount of nucleic RNA between cell subtypes in the deconvolution analysis. Such a task requires single-cell RNA-seq data from a variety of cell types/subtypes.

## Conclusion

Through FGCN module mining, we identified cell-type specific modules that were differentially expressed and/or differentially co-expressed between brains affected by AD and healthy brains in five large cohort studies. Increased expression of a core microglia module in the AD samples can be well explained by an increase in the proportion of microglia cells rather than by upregulation of microglia gene expression. For other cell type specific modules (neuron, astrocyte, endothelia and oligodendrocytes), changes in cell-type composition and regulatory changes each impact module expression levels differently. Investigation of network hub gene changes in AD identified several differentially expressed transcription factors (*BCL6* and *STAT3*) as potential key regulators of transcriptional changes in neuron modules. The expression of astrocyte modules was highly correlated with three AD clinical markers, indicating that the core astrocyte genes in those modules can serve as biomarkers for disease progression. The combination of DE, DC, and cell-type deconvolution analyses provides a powerful approach to delineate the origin of transcriptomic changes in bulk sample data, leading to a deeper understanding of the roles of specific genes in disease progress.

## Methods

### Datasets and sample processing

Five transcriptomic datasets from one or multiple regions of brain tissue from patients with AD and healthy controls were used (Table 1), including two microarray datasets GSE5281^79^, GSE48350^80^ from NCBI Gene Expression Omnibus and three RNA-seq datasets from the Accelerating Medicines Partnership-Alzheimer’s Disease (AMP-AD) Knowledge Portal. The three AMP-AD RNA-seq datasets were designated as MSBB^53^, ROS/MAP^52^, and Mayo^51^ and contained transcriptome-wide FPKM values or raw read counts for AD and control samples. Four additional transcriptomic datasets containing AD and control human brain samples were used for validation of differential gene expression. The sample sizes and other details for all datasets are summarized in Table 1.

The raw microarray datasets were subjected to RMA normalization using the R/Bioconductor package “affy” with default parameters^81^. All datasets were pre-filtered to remove probes without gene annotation. For genes with multiple probes, we selected the probe with the highest expression value^82,83^. For the RNA-seq datasets, we removed genes with more than 50% zero expression levels across samples from each condition (AD or control). Next, for the microarray data and the RNA-seq data, we removed genes with variance in the bottom 20th percentile of the entire dataset. Genes with mean expression value in the bottom 10th percentile were also removed. At the end of the processing, 10,931 genes that were present in all five pre-filtered datasets were used for FGCN mining.

### Frequent gene co-expression network construction and module detection

FGCN modules were mined separately in the AD and control samples. For the microarray datasets, PCC was used as the correlation measure of every gene pair. For the RNA-seq datasets, because of the large range of expression values and non-Gaussian distribution, Spearman correlation coefficient (SCC), which is much less sensitive than PCC to outliers, was used^84^.

Because the range of correlation values varied substantially among the different datasets, instead of applying a uniform threshold on the correlation coefficients across datasets, the gene pairs within the top fifth percentile of |PCC| or |SCC| values with *p* values < 0.05 within each dataset were selected for FGCN mining, as in previous similar studies^82^. For each selected gene pair, the frequency was computed across the five datasets and used as the edge weight for FGCN mining, which is the number of times a specific pair of genes appears in the top fifth percentile of correlation lists across all five datasets divided by the total number of lists (5).

The local maximized Quasi-Clique Merger (lmQCM) algorithm^85^ implemented by the online tool package TSUNAMI^86^ was used to mine the FGCN modules in the AD and control samples. lmQCM allows overlaps between modules and is capable of identifying smaller co-expressed local modules than WGCNA. The lmQCM algorithm takes five parameters (t, λ, γ, β, and minimum module size), which were set as follows: t = 1.0, λ = 1.0, γ = 0.81 (the initial co-expressed gene pair of an FGCN module should be a gene pair showing up in at least four of the five datasets), β = 0.3, and minimum module size = 10. The prefixes “AD” and “N” were used to designate modules mined from AD samples and control samples, respectively.

### Measurement of differential expression in GCN modules between AD and control samples

To determine if a gene module was differentially expressed between AD and control samples within a dataset, we adopted the DE score measure for individual genes, similarly to Lui et al.^87^, and extended it to measure a group of genes in a FGCN module. For a module with m genes from AD and control samples, D denotes the AD group, and N denotes the normal control group. We adopted the widely used absolute t-value from t statistics to quantify the degree of DE. The t-value for a given gene *i* is defined as:

$$\left| {t_{i} } \right| = \frac{{\left| {x_{D} - x_{N} } \right|}}{{\sqrt {\frac{{s_{D}^{2} }}{{n_{D} }} + \frac{{s_{N}^{2} }}{{n_{N} }}} }}$$

where $\underline{x}_{D}$ and $\underline{x}_{N}$ are the mean expression levels in the disease and normal states, $n_{D}$ and $n_{N}$ are samples sizes for the disease and normal states, and $s_{D}$ and $s_{N}$ are the standard deviations of expression levels in the disease and normal states^87^.

To adjust for the different module sizes, we normalized the module DE scores by dividing the sum of the t-values of all genes in a specific module by the module size. The normalized DE score is considered the measure of the overall DE level for the module. A higher DE score indicates a larger overall difference in expression of a module. For each frequent module, we calculated its DE scores in all five datasets and then used the median score as the median DE score for that module.

### Measurement of differential co-expression in GCN modules between AD and control samples

To determine the DC level of a module between two different conditions (AD vs. control) within a dataset, we first computed the DC measure of each pair of genes in the module^87^. For a pair of genes, we used the DC measure Z to quantify the correlation difference between two genes in the AD and normal samples. $Z_{ij}$ between $X_{i}$ and $X_{j}$ is defined as:

$$Z_{ij} = \frac{{\left| {z_{ij}^{N} - z_{ij}^{D} } \right|}}{{\sqrt {\frac{1}{{n_{N} - 3}} + \frac{1}{{n_{D} - 3}}} }}$$

where $n_{D}$ and $n_{N}$ are samples sizes in the normal and AD disease conditions, respectively, and $z_{ij}^{N}$ and $z_{ij}^{D}$ are the Fisher-transforms of the Pearson/Spearman correlation coefficients $r_{ij}^{N}$ and $r_{ij}^{D}$, which were defined as:

$$\begin{aligned} z_{ij}^{N} & = \frac{1}{2}ln\left| {\frac{{1 + r_{ij}^{N} }}{{1 - r_{ij}^{N} }}} \right|, \\ z_{ij}^{D} & = \frac{1}{2}ln\left| {\frac{{1 + r_{ij}^{D} }}{{1 - r_{ij}^{D} }}} \right|. \\ \end{aligned}$$

We calculated $Z$ values for every pair of genes in a given module and then normalized the scores by dividing by the $L_{2}$ norm of $Z$ values within the module. The resulting $L_{2}$ norm of $Z$ values is considered the overall DC measure of the module, termed as the *DC score*. A higher DC score indicates a greater overall DC level in a module between AD and control samples. For each frequent module, we calculated DC scores in all five datasets and then used the median score as the median DE score of that module.

The original z-score as well as the modified DC score only take the absolute value of a correlation coefficient into account. In order to determine if the correlation was stronger or weaker in the AD samples compared with that in the control samples within a module, we measured the correlation change using the CCI^35^. CCI values range from 0 to 1, with larger values indicating stronger correlation between gene expression levels. We plotted the CCI values of a given module across the five datasets to determine if the correlations within the module were stronger or weaker in the AD samples compared with those in the controls. We determined the significance of the differences between the CCI values of the AD samples and controls by t-test.

### Categorizing the modules into high and low DE and DC groups

After defining the DE and DC measures for the modules, we classified the modules into high and low DE/DC categories based on the median DC/DE scores (solid line in Fig. 1a). Then, we partitioned the modules into four categories for further analysis: HDC_HDE, HDC_LDE, LDC_HDE, and LDC_LDE.

### FGCN module functional enrichment analysis

ToppGene^88^ was used to perform GO and pathway enrichment analysis of the FGCN modules. We used all the genes from each module in the analysis. To compare the results of the GO analysis using only 10,931 genes as background, we also conducted GO analysis with DAVID (Database for Annotation, Visualization and Integrated Discovery https://david.ncifcrf.gov/) to check the same categories of GO enrichments (Biological Function, Pathways, and Cellular Component). The results are presented in Supplementary Table 16. Because the GO analysis results from DAVID largely overlapped with the results from ToppGene, we only showed the results from ToppGene. The functional enrichment terms were considered significantly enriched if they had an FDR adjusted *p* value < 0.05. Enriched transcription factors targeting the module genes were obtained from Enrichr with database “TRANSFAC_and_JASPAR_PWMs” with a cutoff for adjusted *p* value of 0.05 (Kuleshov et al., 2016). To check if the module gene members were transcription factors, we compared the gene list against the online database TFcheckpoint^89^.

### Differential gene expression analysis between AD and control samples for individual datasets

We performed DE analysis for each of the two microarray datasets with the R package “limma” and for each of the three RNA-seq datasets with the R package “limma-voom” (built for RNA-seq analysis) ^90^. Foldchange of 1.2 and FDR adjusted *p* value < 0.05 were used as the threshold for differentially expressed gene selection. We only considered genes differentially expressed if they reached the threshold for differential expression in at least two datasets. To confirm the differentially expressed genes, we repeated the DE analysis using three human brain transcriptomic datasets containing AD and control samples from the NCBI GEO database (GSE15222, GSE33000, GSE84422) and one dataset from the Allen Brain Atlas Aging, Dementia, and Traumatic Brain Injury Study (https://aging.brain-map.org/). The results are shown in Table 2. We conducted Fisher’s exact test for the enrichment of DE genes for each FGCN module with *p* < 0.05 as the threshold for significance.

### Brain cell-type deconvolution in each individual dataset

We obtained brain cell type-specific marker genes from McKenzie et al.^32^, who compared and contrasted five human and mouse cell type-specific transcriptome-wide RNA expression datasets to identify consensus brain cell-type marker genes. We estimated the relative proportions of brain cell types in our datasets using the R package “BRETIGEA”^32^ with the marker gene lists from the same package to deconvolute the five major cell types: neurons, microglia, astrocytes, endothelia, and oligodendrocytes. We obtained cell proportion indices from BRETIGEA using the default settings. We analyzed the changes in the proportions of each cell type between AD and control samples by Wilcoxon rank sum test, using false discovery rate-adjusted *p* values < 0.05 or 0.01 as the threshold for significance (*for *p* value < 0.05, **for *p* value < 0.01 in the boxplots).

### FGCN module enrichment of cell type-specific marker genes

We used the marker gene lists for neurons, astrocytes, oligodendrocytes, microglia, and endothelia for cell type-enrichment analysis of the modules in the AD samples and controls (Supplementary Table 1). Enrichment of cell-type markers within modules was assessed by hypergeometric test, with a significant enrichment defined by a false discovery rate-adjusted *p* value < 0.05.

### Analysis of gain or loss of hub genes between AD samples and controls

In a weighted network, the degree of a node, which labels the node strength, is defined as the sum of weights. We examined the gene node-degree distributions of the co-expression networks generated from the highly correlated gene pairs in the five datasets (see the FGCN mining section) and defined the nodes with degrees in the top fifth percentile as hub genes in the network^91^. We compared the hub genes in AD and control networks and defined the hub genes present in the control network but not in the AD network as “lost hub genes” and the hub genes present in the AD network but not in the control network as “gained hub genes.”

### Comparison of GCN connectivity with respect to neuronal cell proportions for the hub genes

Because the identified hub genes mostly had neuronal functions, we focused our analysis of connectivity vs. cell-type proportion on the neuron cell proportions. We divided the samples in the MSBB dataset into two group with high and low neuron proportions, respectively, based on the median values across samples. We compared the GCN connectivity between the high and low groups in the AD samples and in the control samples. The results are shown in Supplementary Fig. 12.

### Computation of module eigengenes and correlation with cell abundance and clinicopathological attributes

We performed principle component analysis on the expression matrices of the identified FGCN modules in the AD and control samples. The first principle component (PC1) of the expression matrix of a specific module or a gene set (in the case of mic1) was computed as the eigengene value of that module using R function prcomp, which represents the weighted average expression profile for the module^82,85^. We tested the association between the expression of each FGCN module in MSBB dataset and clinicopathological attributes by calculating the Spearman correlation between each module’s eigengene profile and three clinicopathological traits, namely, CDR, BB score, and Plaque_Mean. To check the extent to which the change in cell-type proportion affected the module expression level, we also examined the correlation between module eigengene values in all five datasets and the corresponding estimated cell-type relative proportions by Pearson correlation.

### Examination of the neuron proportion changes across different brain regions

We observed that some gene modules specifically enriched with neuron markers showed stronger correlation relationships in the AD samples than in the controls, which is intriguing given the perturbed neuron activities in AD. Because we performed the FGCN module mining with combined data for datasets with multiple brain regions, we suspected that for neuron-associated genes, the detected co-expression relationship might be due to changes in neuron populations either across brain regions or across the cohort. Therefore, we checked for concordant cell population changes in different brain regions separately in the AD samples and in the controls. Specifically, we examined whether the estimated neuron or microglia population changes correlated differently across brain regions in the AD and control samples. For that, we used the MSBB dataset, because it contains matched samples of four brain regions from 56 patients with AD patients and 40 healthy controls. For each condition (AD and control), we computed the CCI value that indicates the overall correlation of relative cell-type proportions across the four brain regions. We compared the CCIs of the neuron/microglia relative proportions across the AD samples with that across the control samples. A higher CCI for the AD samples in comparison with the controls would suggest that changes in cell-type proportions in specific brain regions were the cause of the strong co-expression within the neuron modules in the AD samples, whereas a lower CCI for the AD samples would suggest that regulatory changes could be the cause of the strong co-expression within the neuron modules.

### Identification of genes regulated by enriched transcription factors in the neuron modules

To identify genes regulated by the transcription factors in Table 2, we obtained ChIP-seq data for BCL6 and STAT3 from previous studies^41,42^. We then overlapped the peak regions in those data with our AD1 and N1 module gene list. The full list of genes with BCL6/STAT3 peaks in their promoter regions is given in Supplementary Table 12.

## Supplementary Information

Supplementary Information.

Supplementary Table 2.

Supplementary Table 3.

Supplementary Table 4.

Supplementary Tables 5, 6.

Supplementary Table 7.

Supplementary Tables 8, 9, 10.

Supplementary Table 11.

Supplementary Table 12.

Supplementary Table 13.

Supplementary Table 14.

Supplementary Table 15.

Supplementary Table 16.

Supplementary Table 17.

Supplementary Table 18.

## Data Availability

Only publicly available data were used in this study. All major analysis results are attached in submission and available for public use. Further requests can be fulfilled by contacting the corresponding authors. The authors declare that they have no competing interests. This article has been deposited to the pre-peer-review online system Research Square and can be accessed on https://www.researchsquare.com/article/rs-26385/v1. This article is not published nor is under consideration for publication elsewhere.
