# Supplementary material for: Combinatorial analyses reveal cellular composition changes have different impacts on transcriptomic changes of cell type specific genes in Alzheimer’s Disease
Source: Sci Rep. 2021 Jan 11;11:353. doi: 10.1038/s41598-020-79740-x (PMC7801680; doi:10.1038/s41598-020-79740-x)
Supplement: Supplementary file 1 — Supplementary Information. [file 41598_2020_79740_MOESM1_ESM.docx]

**Supplementary Figures and Table legends for**

**Combinatorial analyses reveal cellular composition changes have different impacts on transcriptomic changes of cell type specific genes in Alzheimer’s Disease**

Travis S. Johnson^#1^, Shunian Xiang^#2,3^, Tianhan Dong^4^, Zhi Huang^5^, Michael Cheng^2^, Tianfu Wang^3^, Kai Yang^6^, Dong Ni*^3^, Kun Huang*^7^, Jie Zhang*^1,8^

^1^Department of Biostatistics, Indiana University, School of Medicine, School of Medicine, Indianapolis, IN 46202, USA.

^2^Department of Medical & Molecular Genetics, Indiana University, School of Medicine, Indianapolis, IN 46202, USA.

^3^Guangdong Key Laboratory for Biomedical Measurements and Ultrasound Imaging, School of Biomedical Engineering, Shenzhen University, Shenzhen 518060, China.

^4^Department of Pharmacology, Indiana University, School of Medicine, Indianapolis, IN46202, USA;

^5^Department of Electrical and Computer Engineering, Purdue University, West Lafayette, IN

47907, USA.

^6^Department of Pediatrics, Indiana University, School of Medicine, Indianapolis, IN 46202, USA.

^7^Department of Medicine, Indiana University, School of Medicine, Indianapolis, IN 46202, USA.

^8^Lead Contact

*Corresponding authors: Jie Zhang: jizhan@iu.edu; Kun Huang: kunhuang@iu.edu; Dong Ni: [nidong@szu.edu.cn](mailto:nidong@szu.edu.cn)

# these author contribute equally.

**Supplementary Table legends**

**Table 1.** All of the FGCN modules are classified into four groups based on the DC and DE scores and their characteristics.

**Table 2.** FGCN module genes from AD/Normal brain samples and the overlap (Jaccard index) between each pair of AD and normal control FGCN modules. (sup-Table2.xlsx)

**Table 3.** DE and DC score summary for all AD/Normal control FGCN modules. (sup-Table3.xlsx)

**Table 4.** Top 10 enriched GO/pathway terms of each AD/normal control FGCN module. (sup-Table4.xlsx)

**Table 5-6.** Frequently down/up expressed genes (in at least two datasets) between AD and control samples, merged for all five datasets. (sup-Table5-6.xlsx)

**Table 7.** Enrichment of gene markers in five major cell types in all AD/normal control FGCN modules. (sup-Table7.xlsx)

**Table 8.** Enriched pathway/GO terms for overlapping genes of AD1 and N1 using ToppGene. (sup-Table8-10.xlsx)

**Table 9.** Enriched pathway/GO terms in genes uniquely to AD1 using ToppGene. (sup-Table8-10.xlsx)

**Table 10.** Enriched pathway/GO terms in genes uniquely to N1 using ToppGene. (sup-Table8-10.xlsx)

**Table 11.** Enriched transcription factors and their targets for each FGCN modules. (sup-Table11.xlsx)

**Table 12.** Transcription factor BCL6/STAT3 targeted genes in AD1 and N1(sup-Table12.xlsx).

**Table 13.** The overlap of AD3/N6 microglia module with two previously identified disease associated microglia modules (sup-Table13.xlsx).

**Table 14**. The full 547 hub gene lists for AD samples and normal control samples combined for all five datasets and the overlaps with neuron module AD1 and N1 genes.

**Table 15.** The Correlation of FGCN module eigengene and cell types with clinicopathological measurements and associated p values in MSBB dataset. (sup-Table15.xlsx)

**Table 16.** The gene ontology enrichment analysis on FGCN modules using DAVID and 10,931 genes are background.

**Table 17.** AD vs. control differential gene expression analysis on GSE48350 expression after BRETIGEA adjustment for cell proportion change.

**Table 18.** Eigengene correlation of AD3 and N3 in AD and control cohorts, and the correlation of each eigengene to proportion of microglia population in five datasets.

**Supplementary Table -1**

**Summary of the characteristics of all FGCN modules from AD and healthy control samples.**

| **Module category & name** | **Module size** | **Up-reg. genes in AD vs. Healthy** | **Down-reg. genes in AD vs. Healthy** | **Enriched cell type** |
| --- | --- | --- | --- | --- |
| **HDE_HDC** |  |  |  |  |
| AD1 | 1,247 | 29 | 593 | neuron |
| AD2 | 197 | 159 | 0 | astrocyte |
| AD7 | 21 | 17 | 0 | oligodendrocyte |
| AD11 | 14 | 12 | 0 | - |
| N1 | 1,003 | 10 | 449 | neuron |
| N2 | 66 | 1 | 31 | neuron |
| N5 | 25 | 23 | 0 | astrocyte |
| N8 | 13 | 0 | 9 | - |
| **HDE_LDC** |  |  |  |  |
| N6 | 21 | 19 | 0 | microglia |
| AD3 | 38 | 33 | 0 | microglia |
| AD14 | 10 | 9 | 0 | endothelia |
| N9 | 12 | 2 | 0 | - |
| **LDE_HDC** |  |  |  |  |
| AD5 | 29 | 8 | 0 | oligodendrocyte |
| AD8 | 21 | 21 | 0 | endothelia |
| AD9 | 21 | 0 | 0 | - |
| AD12 | 11 | 9 | 0 | oligodendrocyte |
| **LDE_LDC** |  |  |  |  |
| AD4 | 32 | 0 | 0 | oligodendrocyte |
| AD6 | 29 | 20 | 0 | oligodendrocyte |
| AD10 | 17 | 0 | 0 | - |
| AD13 | 10 | 9 | 0 | endothelia |
| AD15 | 10 | 0 | 0 | - |
| N3 | 47 | 26 | 0 | oligodendrocyte |
| N4 | 35 | 25 | 0 | oligodendrocyte |
| N7 | 19 | 0 | 0 | - |

**Supplementary figures**

**Figure 1** Top 10 enriched pathways of the two microglia modules combined.


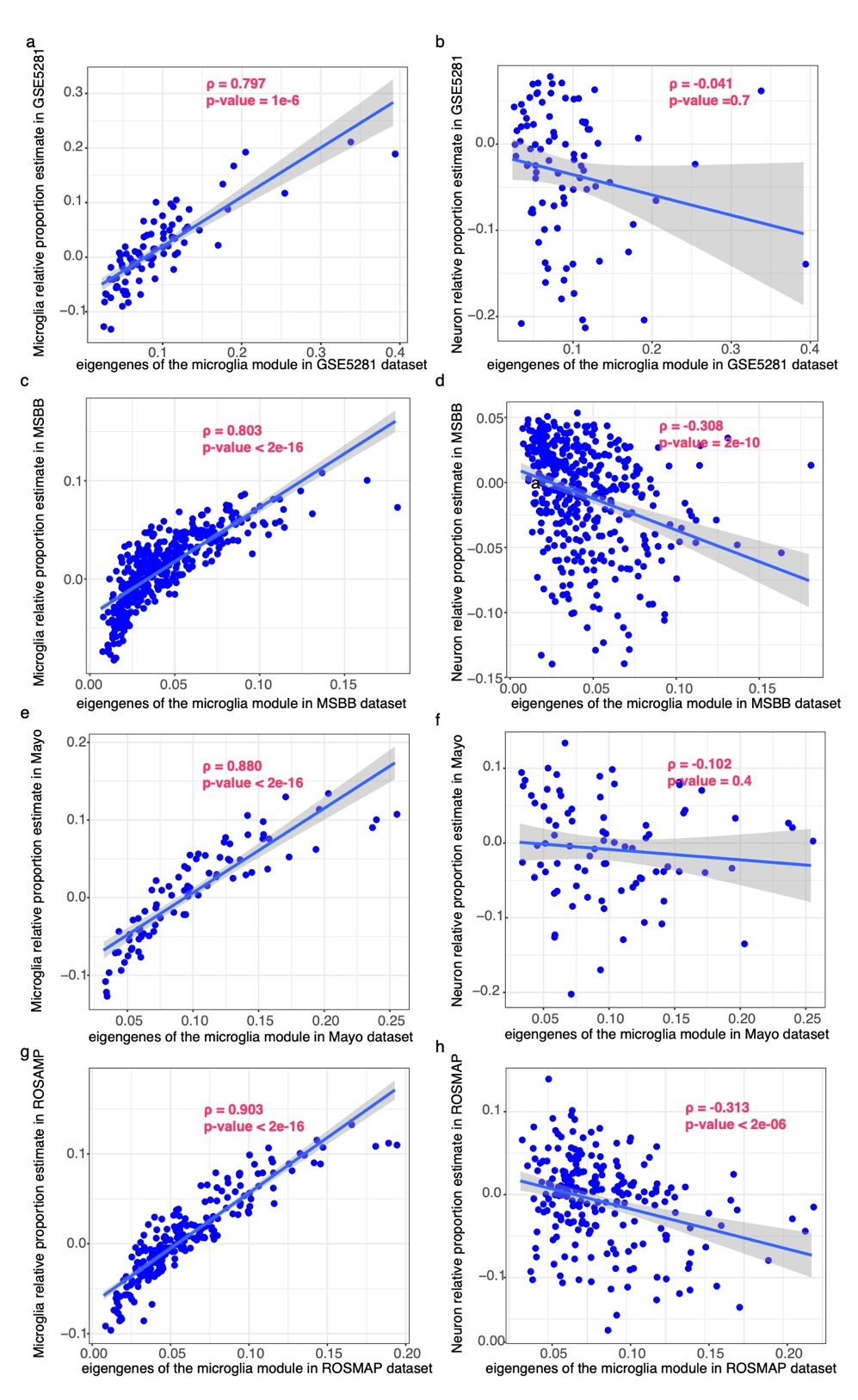


**Figure 2** The Pearson correlation of the combined microglia module expression (eigengene values) with the estimated microglia cell proportions vs. with the estimated neuron cell proportions in GSE5281, MSBB, Mayo and ROSMAP datasets. a. Correlation with the estimated microglia cell proportions in GSE5281 dataset. b. Correlation with the estimated neuron cell proportions in GSE5281 dataset (control). c. Correlation with the estimated microglia cell proportions in MSBB dataset. d. Correlation with the estimated neuron cell proportions in MSBB dataset (control). e. Correlation with the estimated microglia cell proportions in Mayo dataset. f. with the estimated neuron cell proportions in Mayo dataset (control). g. Correlation with the estimated microglia cell proportions in ROSMAP dataset. h. Correlation with the estimated neuron cell proportions in ROSMAP dataset (control). Gray area indicates the area between the 95% confidence intervals. Figure b, d, f, h are shown as contrast to a, c, e, g with low correlations.


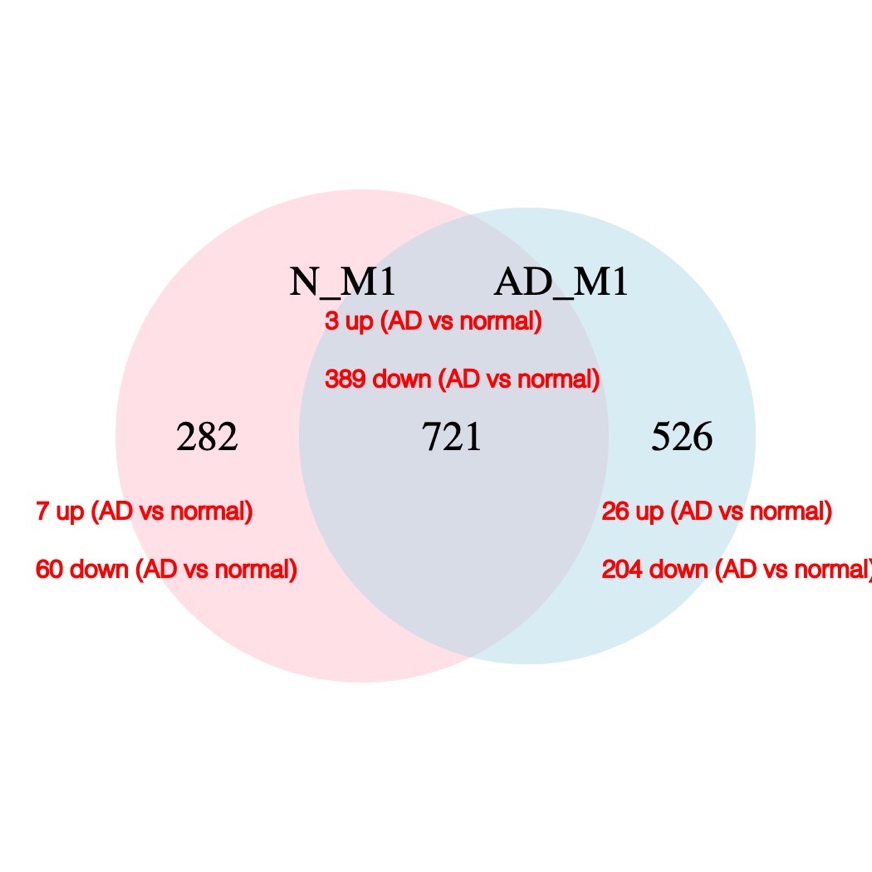


**Figure 3** Overlapping of the neuron modules AD1 and N1, and the differentially expressed genes (in at least two datasets) in each compartment (red labels).


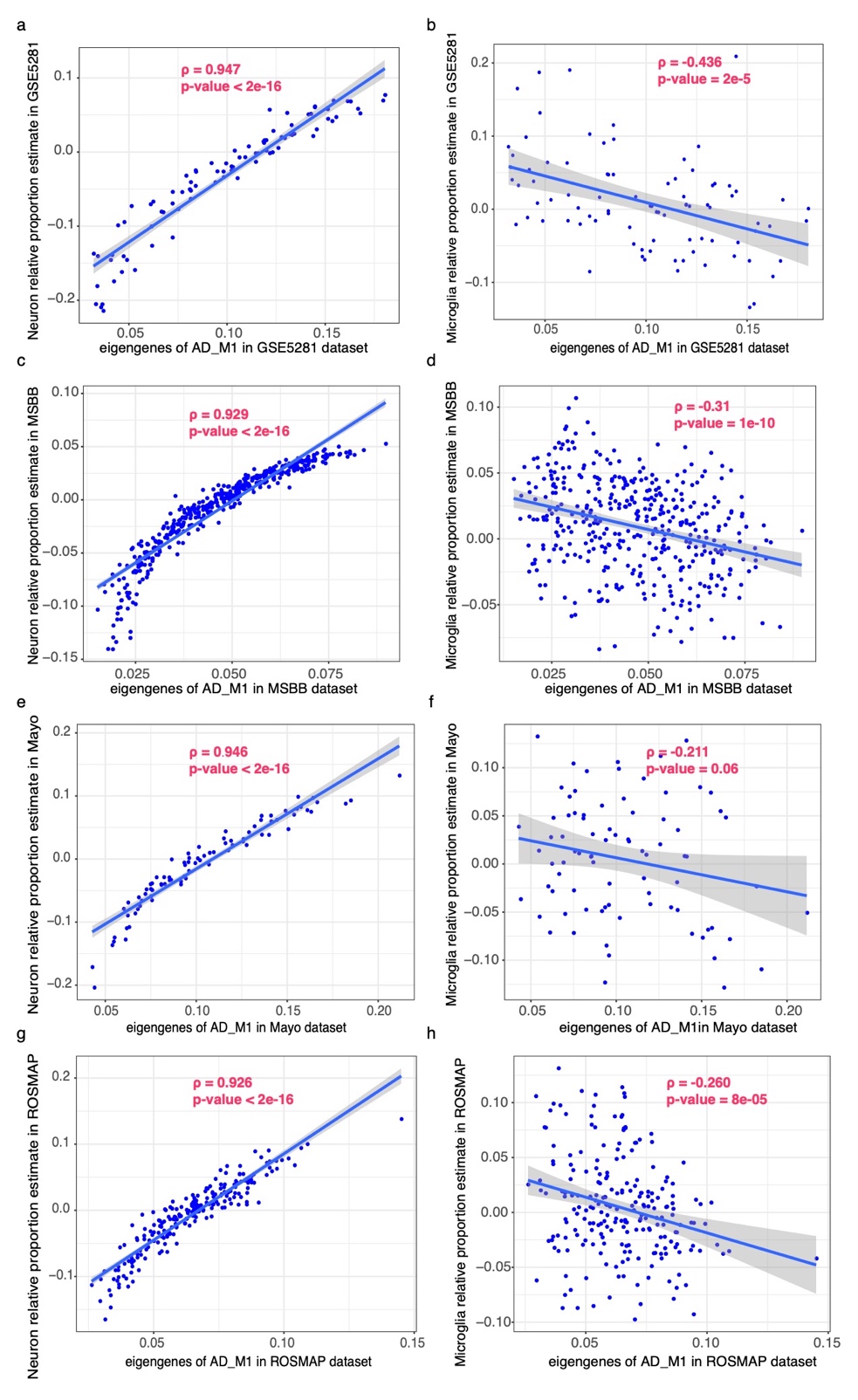


**Figure 4** The Pearson correlation of neuron module AD1 expression (eigengene values) with the estimated neuron cell proportions vs. with the estimated microglia cell proportions in GSE5281, MSBB, Mayo and ROSMAP datasets. a. Correlation with the estimated neuron cell proportions in GSE5281 dataset. b. Correlation with the estimated microglia cell proportions in GSE5281 dataset (control). c. Correlation with the estimated neuron cell proportions in MSBB dataset. d. Correlation with the estimated microglia cell proportions in MSBB dataset (control). e. Correlation with the estimated neuron cell proportions in Mayo dataset. f. Correlation with the estimated microglia cell proportions in Mayo dataset (control). g. Correlation with the estimated microglia cell proportions in ROSMAP dataset. h. Correlation with the estimated microglia cell proportions in ROSMAP dataset (control). Gray area indicates the area between the 95% confidence intervals.


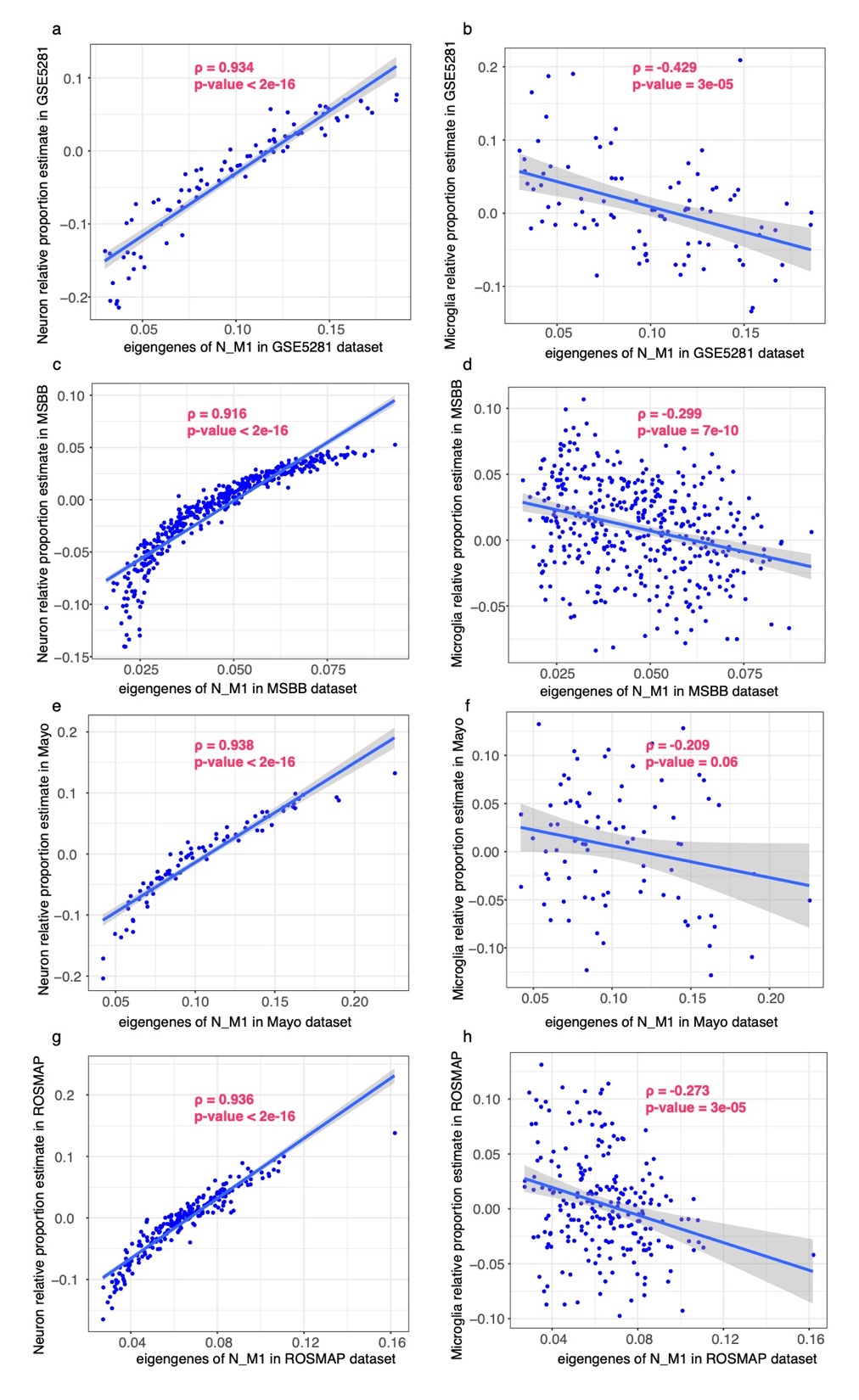


**Figure 5** The Pearson correlation of the expression of neuron module N1 with the estimated neuron cell proportions vs. with the estimated microglia cell proportions in GSE5281, MSBB, Mayo and ROSMAP datasets. a. Correlation with the estimated neuron cell proportions in GSE5281 dataset. b. Correlation with the estimated microglia cell proportions in GSE5281 dataset (control). c. Correlation with the estimated neuron cell proportions in MSBB dataset. d. Correlation with the estimated microglia cell proportions in MSBB dataset (control). e. Correlation with the estimated neuron cell proportions in Mayo dataset. f. Correlation with the estimated microglia cell proportions in Mayo dataset (control). g. Correlation with the estimated neuron cell proportions in ROSMAP dataset. h. Correlation with the estimated microglia cell proportions in ROSMAP dataset. Gray area indicates the area between the 95% confidence intervals.


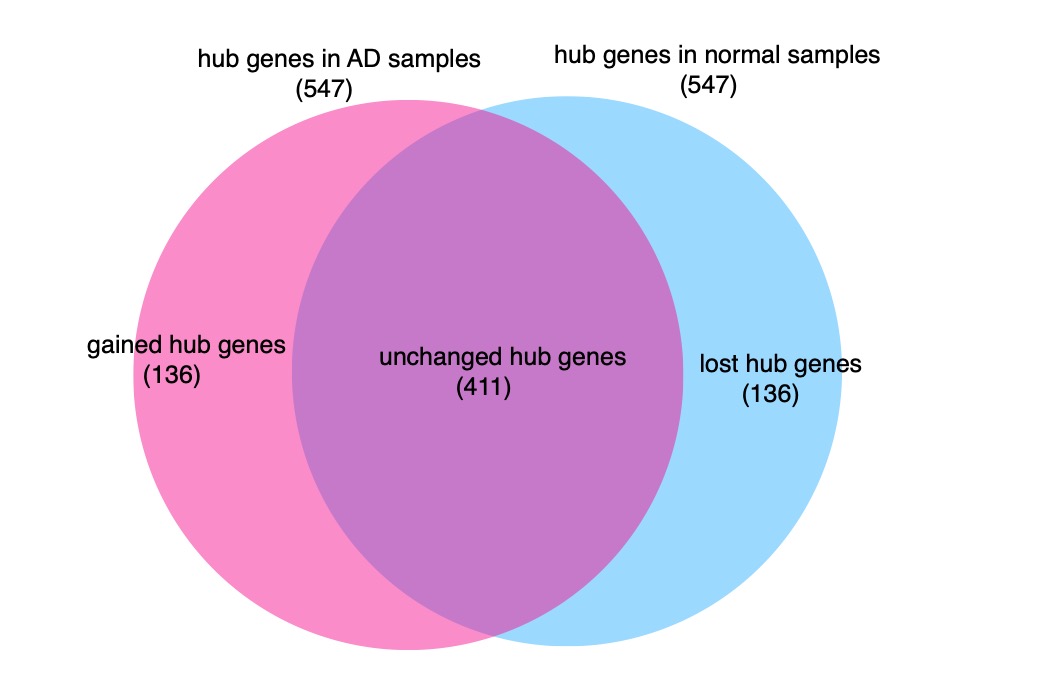


**Figure 6** 547 hub genes from the frequent co-expression networks in AD vs. in normal samples (top 5 percentile connections in each group). The pink area denotes the gained hub genes in AD FGC network, the blue area denotes the lost hub genes in AD network but show up in normal network, and the purple area denotes the unchanged hub genes.

**
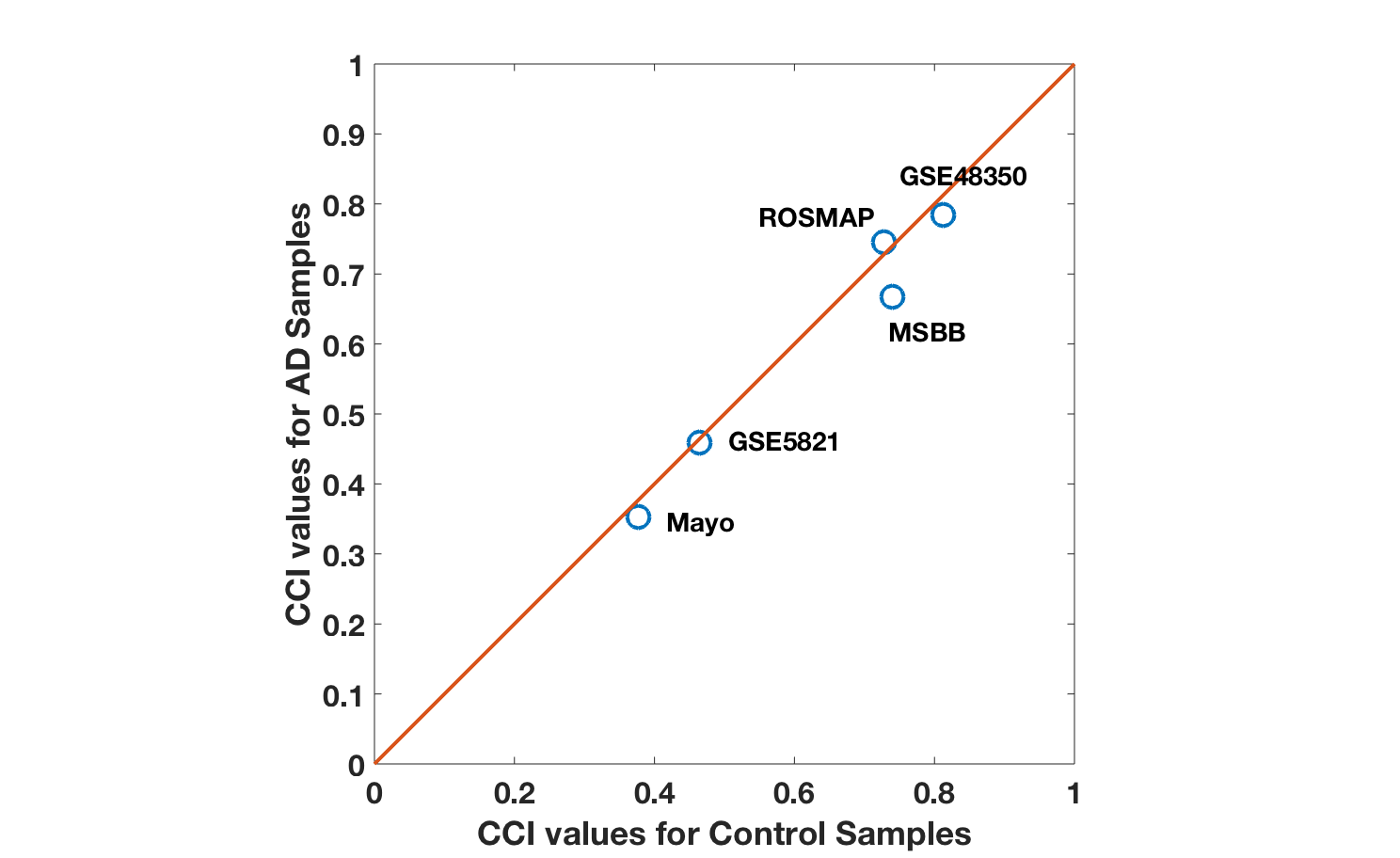
**

**Figure 7** The scatterplot for the overall expression correlation (as measured by CCI) for the 13 DAM genes from AD3/N6 microglia module across the five datasets. The alignment to the diagonal line indicates relatively unchanged correlation for this subset of genes.

**Figure 8** RNA-seq expression of BCL6 and STAT3 among six major cell types in healthy human brains (Data generated using BrainRNAseq.org).

**A B**

**C D**

**Figure 9** Plots of mean CCI across five datasets for HDE_HDC modules that are not shown in main figure: A) AD2; B) AD7; C) AD14; D) N5. These plots show the CCI values are not as aligned to the diagonal line as in microglia modules AD3+N6 in Figure 3, indicating more apparent co-expression change in between AD and control samples.

**Figure 10** Cell proportion estimate in AD samples vs. module eigengene for AD2,7,14 and N5. Column 1: AD2 astrocyte module on five datasets. Column 2: AD7 oligodendrocyte module on five datasets. Column 3: AD14 endothelia module on five datasets. Column 4: N5 astrocyte module on five datasets. The PCC values, p value and confidence intervals are shown on top of each plot.

**A B**

**C D**

**Figure 11** Mean cell proportion estimate change vs. DE scores for AD2,7,14 and N5 module across five datasets. A: AD2 astrocyte module; B: AD7 oligodendrocyte module; C: AD14 endothelia module; D: N5 astrocyte module.

**A**


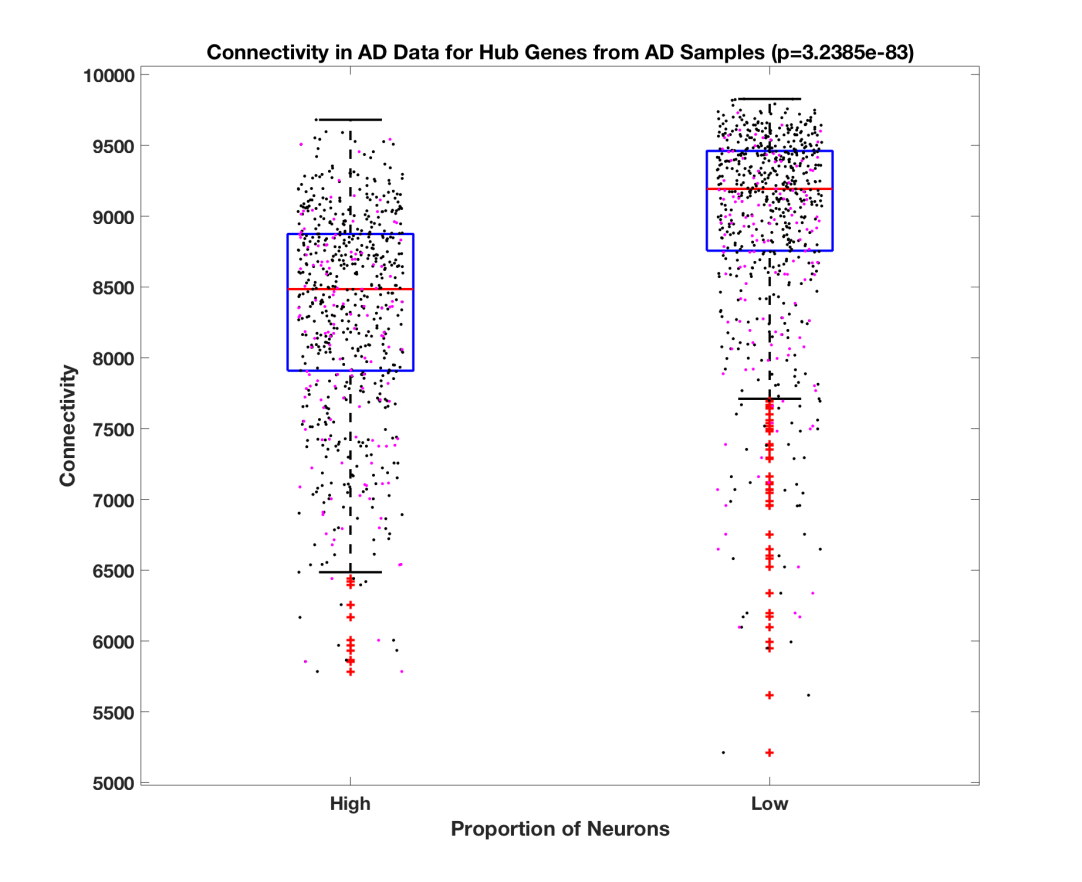


**B**

**
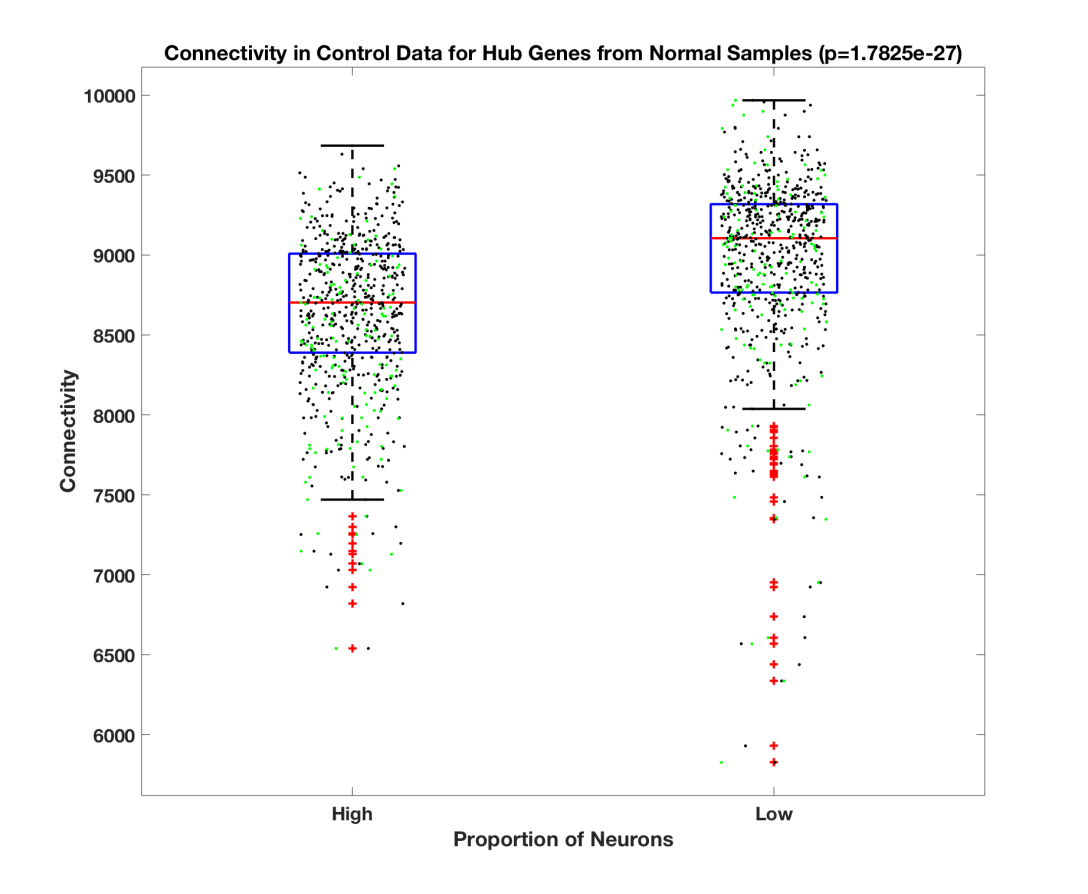
**

**Figure 12** Connectivity comparisons between neuron cell proportion high and low group for the two 547 hub gene sets from AD and control GCN network. A: AD hub genes in AD samples of MSBB dataset. AD gained hub genes are highlighted with magenta. B: Normal control hub genes in control samples of MSBB dataset. Control gained hub genes are highlighted with green.

**A**

**B**

**Figure 13** Synthetic eigengene/module using AD-associated microglia subtype genes mic1 cluster from Marthys et al. 2019 *Nature* publication and its correlation to cell proportion estimate as well as to DE score. A: Cell proportion estimate vs. mic1 eigengene in AD samples of GSE48350. B: Mean microglia proportion change vs. DE scores in five datasets for mic1 genes.
